# Physical characteristics comparison between maytansinoid-based and auristatin-based antibody-drug conjugates

**DOI:** 10.37349/etat.2021.00064

**Published:** 2021-12-31

**Authors:** Tomohiro Fujii, Calliste Reiling, Colette Quinn, Michal Kliman, Brian A. Mendelsohn, Yutaka Matsuda

**Affiliations:** 1 Ajinomoto Bio-Pharma Services, 11040 Roselle Street, San Diego, CA 92121, United States; 2 TA Instruments, 159 Lukens Drive, New Castle, DE 19720, United States; 3 Waters Corporation, 34 Maple Street, Milford, MA 01757-3696, United States; 4 Exelixis Inc, 1851 Harbor Bay Pkwy, Alameda, CA 94502, United States; Institute of Oncology Research, Switzerland

**Keywords:** Antibody-drug conjugates, hydrophobicity, drug-linker, conjugation

## Abstract

**Aim:**

Direct analytical comparison of two major drug-linkers in the antibody-drug conjugate (ADC) field was conducted.

**Methods:**

Four different analytical methods [AlogP calculation, reverse phase (RP) high-performance liquid chromatography (HPLC; RP-HPLC), size exclusion chromatography HPLC (SEC-HPLC), and differential scanning calorimetry (DSC)] were tested for this comparison.

**Results:**

Maytansinoid-based ADCs showed less hydrophobicity than auristatin-based ADCs. Regardless of the drug-linker and drug-to-antibody ratios (DARs), the stability detected by DSC was decreased by conjugation.

**Conclusions:**

The cost and time-efficient analytical comparison described in this manuscript may be useful information for an initial characterization of ADCs prior to detailed biological studies.

## Introduction

Antibody-drug conjugates (ADCs) are a promising biopharmaceutical modality in the oncology field due to the selectivity against their target [1–4]. Eleven ADCs have been approved for clinical use by the Food and Drug Administration. These bioconjugates are produced via linking small molecules (drug-linkers) to monoclonal antibodies possessing a binding affinity for a tumor-associated target antigen in a specific manner.

**Figure 1.**
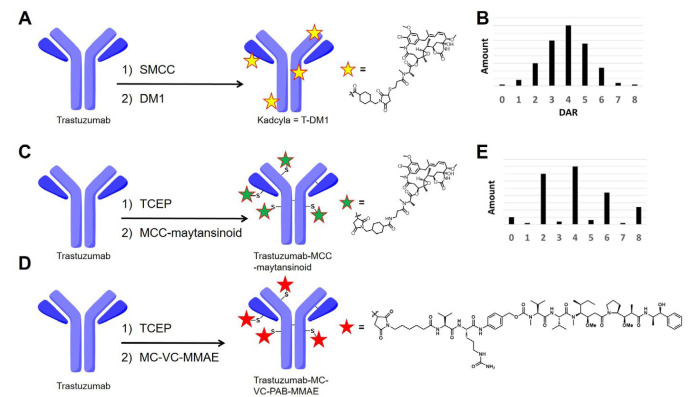
Summary of conjugation chemistry to produce ADCs. A. Native lysine conjugation to synthesize T-DM1 (kadcyla); B. DAR distribution of lysine-based ADCs; C. native cysteine conjugation to synthesize trastuzumab-MCC-maytansinoid; D. native cysteine conjugation to synthesize trastuzumab-(MC)-(VC)-MMAE; E. DAR distribution of cysteine-based ADCs. SMCC: succinimidyl-4-(*N*-maleimidomethyl)cyclohexane-1-carboxylate; TCEP: Tris(2-carboxyethyl)phosphine; MC: maleimidocaproyl; VC: valine-citrulline; MCC: (*N*-maleimidomethyl)cyclohexane-1-carboxylate; PAB: p-aminobenzoyloxycarbonyl; MMAE: monomethyl auristatin-E; DAR: drug-to-antibody ratio

The key factor for ADC efficacy is the choice of the drug-linker compounds. These small cytotoxic molecules are familiar to traditional synthetic organic chemists [5]. A wide variety of natural products have been reported, some of which have the potential to be used as payloads. However, this tremendous natural products toolbox has not been utilized effectively in the field of ADCs and currently, only a handful of natural compound analogs have been applied successfully to ADCs [6]. Maytansinoids and auristatins are classes of cytotoxic compounds both of which are well-used payloads for ADCs in the clinical trial phase [7]. DM1, one of the maytansinoids, is a payload of trastuzumab emtansine (T-DM1; general name: kadcyla) and other maytansinoid-based ADCs are currently in the clinical phase. T-DM1 was produced by native lysine chemistry which conjugates trastuzumab with DM1 in the stochastic manner [2]. The synthesis of these ADCs can result in a very large number of species because the antibodies have about 80 exposed and reactive lysine residues available for conjugation (Figure 1A, 1B).

MMAE, an auristatin-class compound, is widely used in ADCs that are currently in clinical trials [8]. MMAE-based ADCs are commonly synthesized by native cysteine conjugation chemistry producing “semi-random” ADCs (Figure 1C–1E). Reduction of the interchain cysteines followed by thiolmaleimide conjugation afford relatively lower heterogeneous ADCs than native lysine conjugation [9]. From this advantage, native cysteine conjugation successfully provided six commercially approved ADCs (Adcetris, Polivy, Padcev, Blenrep, Zynlonta, and Tivdak) and is used for many ADCs in clinical trials. These clinical successes clearly indicate that native cysteine conjugation is a viable and clinically relevant option for ADC synthesis.

Interestingly, both maytansinoids and auristatins, two of the most validated payload classes in ADCs, are tubulin inhibitors, but their mode of action is slightly different. Maytansinoids target the maytansine site of microtubes, however, auristatins (including MMAE) bind the vinca alkaloid site [7]. Binding these payloads to each target promotes microtube depolymerization.

Even though both maytansinoids and auristatins are well-established payload classes, there is a lack of direct comparison available because these payloads are usually conjugated using distinct conjugation chemistries [10, 11]. This prompted us to conduct a comparison study between a maytansinoid and MMAE to examine the potential differences in their physical characteristics. First, to adapt the conjugation chemistry for MC-VC-PAB-MMAE, a cysteine reactive maytansinoid termed MCC-maytansinoid was prepared (ADC structure is shown in Figure 1C and chemical structure of a payload is described in Figure S1). The hydrophobicity of MCC-maytansinoid and MC-VC-PAB-MMAE were compared by calculated LogP and relative retention time via reverse phase (RP) high-performance liquid chromatography (HPLC; RP-HPLC). Native cysteine conjugation provided both the maytansinoid-based ADC and MMAE-based ADC from the anti-human epidermal growth factor receptor 2 (HER2) antibody trastuzumab. Furthermore, differential scanning calorimetry (DSC) analysis and size exclusion chromatography (SEC) analysis of these ADCs showed physical characteristics differences. These analytical methods also compared the stability of lysine-based and cysteine-based ADCs.

## Materials and methods

### Materials

Human IgG1 trastuzumab (Herceptin^®^) was purchased from Roche Pharmaceutical Company (Switzerland). MCC-maytansinoid (Catalog No.: TCRS-1262) was purchased from Abzena (USA). MC-VC-PAB-MMAE (CAS#: 646502-53-6) was purchased from NJ Biopharmaceuticals LLC (USA). All other chemical reagents were acquired from Sigma-Aldrich (USA).

### Calculation of AlogP for drug-linkers

AlogP calculation was conducted by Biovia Draw based on the basic protocol [12].

### Conjugation procedure for trastuzumab-based ADCs with c.a. DAR = 4.0

For initial preparation of the ADC, 2.35 equiv. of TCEP hydrochloride (TCEP-HCl) was added to a solution of trastuzumab (1.0 mg) in conjugation buffer [0.25 mL, pH 7.5, 50 mmol/L PBS, 10 mmol/L ethylenediaminetetraacetic acid (EDTA)]. This reaction mixture was stirred mildly for 2.5 h at room temperature (rt). Dimethylacetamide (DMA, 8% v/v) and 7 eq. of drug-linker were sequentially added to the resulting reaction mixture and stirred mildly for 1 h at rt. The unreacted drug linker was quenched with the addition of 25 eq. of *N*-acetyl-*L*-cysteine (NAC) and mixed for 25 min at rt. The final mixture was purified using NAP-10 desalting columns (purchased from GE Healthcare Life Sciences, USA) and eluted with pH 5.2, 20 mmol/L histidines, 5% trehalose [13].

### Conjugation procedure for trastuzumab-based ADCs with c.a. DAR = 2.0

To obtain ADC with c.a. DAR = 2.0, reduction by 1.1 equiv. of TCEP was conducted at 4°C for 2 h. Following this step, the synthetic procedure for DAR = 4.0 ADC synthesis was followed as described above.

### RP-HPLC analysis to estimate hydrophobicity of drug-linkers

This HPLC analysis was performed on a Sepax Proteomix RP-1000 5 μm 2.1 × 50 mm column (Sepax Technologies, Inc., USA), connected to an Agilent 1260 HPLC system containing a binary gradient pump, temperature-controlled column compartment, autosampler, and a diode array detector. The equipment conditions were as follows: mobile phase A (MPA) = 0.1% trifluoroacetic acid (TFA) and 2% acetonitrile in water; mobile phase B (MPB) = 0.1% TFA in acetonitrile; flow rate = 0.5 mL/min; column temperature = 80°C; sampler temperature = 4°C. Each drug-linker (0.02 mmol/L DMA solution, 5 μL) was injected into the system and eluted over a 22 min run consisting of a 1 min isocratic hold at 30% MPB, a 15 min linear gradient from 30% to 45% MPB, a 3 min wash using 95% MPB, and a 3 min re-equilibration at 30 % MPB. The absorbance was monitored at 280 nm (reference wavelength at 450 nm) [14].

### Pretreatment for RP-HPLC analysis of ADCs

The reductive pretreatment was performed based on previously reported literature [15].

### RP-HPLC analysis for reduced ADCs

This HPLC analysis was performed on an AdvanceBio RP-mAb Diphenyl, 2.1 × 100 mm, 3.5 μm column (Agilent, USA), connected to an Agilent 1260 HPLC system containing a binary gradient pump, temperature-controlled column compartment, autosampler, and a diode array detector. The equipment conditions were as follows: MPA = 0.1% TFA and 2% acetonitrile in water; MPB = 0.1% TFA in acetonitrile; flow rate = 0.4 mL/min; column temperature = 70°C; sampler temperature = 4°C. Each ADCs (0.66 mg/mL, 20 μL) was injected into the system and eluted over a 35 min run consisting of a 2 min isocratic hold at 30% MPB, a 22 min linear gradient from 30% to 48% MPB, a 3 min wash using 95% MPB, and an 8 min re-equilibration at 30% MPB. The absorbance was monitored at 280 nm (reference wavelength at 450 nm) [15].

### SEC analysis of ADCs

ADC samples at 5.0 mg/mL (in 20 mmol/L histidine, 5% trehalose at pH 5.2) were prepared. All the samples were stored at 4°C for 30 days. Then, aggregation analysis was conducted by SEC. The SEC data was acquired an Acquity UPLC BEH200 SEC 300 Å, 4.6 × 300 mm, 1.7 μm column (Waters Corporation). Each sample (1 mg/mL, 40 μL) was injected into the system and ran at 0.25 mL/min at 30°C over a 20 min isocratic hold at 100 mmol/L NaHPO_4_/NaH_2_PO_4_, 250 mmol/L NaCl, 10% v/v isopropanol mobile phase. The absorbance was monitored at 280 nm (reference wavelength at 450 nm).

### DSC analysis of ADCs

ADC samples at 0.5 mg/mL (in 20 mmol/L histidine, 5% trehalose at pH 5.2) were run in duplicate on a Nano DSC (TA Instruments-Waters LLC) equipped with an autosampler and 96-well plates. The structure stability changes in the ADC sample were measured thermodynamically over the temperature range from 15°C to 100°C at a temperature scan rate of 1°C/min. All samples were degassed prior to loading the 96-well plate. A complete thermodynamic profile for the unfolding of each sample was calculated and visualized using NanoAnalyze^TM^ (TA Instruments-Waters LLC). For each analysis, 550 μL of ADC was loaded into the sample well and an equivalent volume of the provided buffer was loaded into the reference side. The instrument was set to scan twice from 15°C to 100°C at 1.0°C/min with a 600-second equilibration. Each sample was run in duplicate and negative control, a buffer-buffer scan was used as the background. A cleaning method of 5 mL detergent followed by 40 mL of water was automatically delivered to the sample and reference cells between each experiment.

All data were evaluated with the NanoAnalyze Software package. A fourth-order polynomial was used as the baseline with two nodes prior to the initial event, one node at the valley between the CH2 and Fab unfolding event, and two nodes were placed above the final unfolding event. Typically, two Gaussian models were applied to the data. More were applied if the error was deemed significant due to asymmetry in the unfolding.

## Results

### Comparison of hydrophobicity of drug-linkers

To understand the hydrophobic difference of both drug-linkers, two different approaches were used. The AlogP value calculated for MC-VC-PAB-MMAE (4.79) was greater than that of MCC-maytansinoids (3.76), which indicates MC-VC-PAB-MMAE is more hydrophobic in nature. The results from RP-HPLC analysis confirm MC-VC-PAB-MMAE is more hydrophobic than MCC-maytansinoid due to the slower elution time of MC-VC-PAB-MMAE 11.5 min compared to MCC-maytansinoid (5.5 min) (Table 1).

**Table 1.** Comparison of each drug-linkers

**Drug-linker**	**AlogP**	**Retention time in HPLC**
MCC-maytansinoid	3.76	5.5 min
MC-VC-PAB-MMAE	4.79	11.5 min

### Preparation of ADCs

Each ADC was prepared by traditional cysteine-based conjugation methodology [13]. This method includes a partial reduction of the interchain disulfide bonds of the antibody by TCEP, followed by thiol maleimide coupling with each drug-linkers. Conjugation with MCC-maytansinoid provided trastuzumab-MCC-maytansinoid with DAR = 4.1. MC-VC-PAB-MMAE showed a similar DAR value to MCC-maytansinoid resulting in trastuzumab-MC-VC-PAB-MMAE with DAR = 4.0. Each DAR was calculated by RP-HPLC analysis under reduced conditions (Table 2). Antibody light chain conjugated with one MMAE compound was eluted later than light chain conjugated with one maytansinoid compound. Conjugates derived from antibody heavy chains (HCs) showed the same phenomenon as light chain derivatives.

**Table 2.** Comparison of retention time of DAR species in RP-HPLC

**DAR species**	**Trastuzumab-MCC-maytansinoid**	**Trastuzumab-MC-VC-PAB-MMAE**
LC	7.0 min	7.0 min
LC + drug-linker	8.2 min	9.6 min
HC	10.2 min	10.2 min
HC + drug-linker	11.0 min	12.1 min
HC + 2 drug-linkers	13.1 min	15.4 min
HC + 3 drug-linkers	14.4 min	18.0 min

LC: antibody light chain; LC + drug-linker: LC conjugated with one drug-linker; HC + drug-linker: HC conjugated with one drug-linker

Additionally, we also synthesized trastuzumab-MC-VC-PAB-MMAE with DAR = 1.9 by mild reduction conditions (reaction temperature: 4°C) [13].

### SEC analysis

To assess the long-term stability of three different ADCs, an SEC analysis was conducted. After 30 days under 4°C all ADCs contained acceptable aggregation levels (less than 2%) (Table 3).

**Table 3.** SEC analysis of three different ADCs

**ADCs**	**Aggregation**
Trastuzumab	0.4%
T-DM1	1.4%
Trastuzumab-MCC-maytansinoid (DAR = 4.1)	0.7%
Trastuzumab-MC-VC-PAB-MMAE (DAR = 2.0)	0.5%
Trastuzumab-MC-VC-PAB-MMAE (DAR = 4.0)	0.5%

### DSC analysis

**Figure 2.**
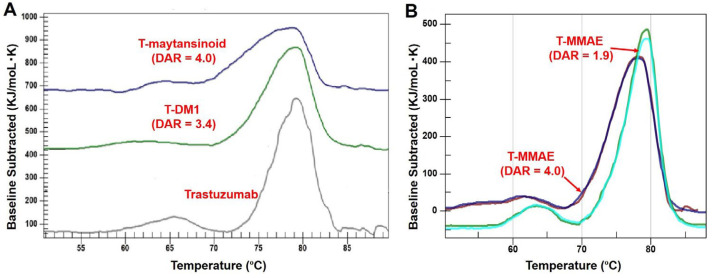
Comparison of DSC thermograms. A. Trastuzumab, T-DM1 and trastuzumab-MCC-maytansinoid (T-maytansinoid); B. trastuzumab-MC-VC-PAB-MMAE (T-MMAE) with DAR = 1.9 and Trastuzumab-MC-VC-PAB-MMAE (T-MMAE) with DAR = 4.0

The thermal stability of trastuzumab and three different ADCs in 20 mmol/L histidines, 5% trehalose at pH 5.2 buffer at 0.5 mg/mL was assessed using DSC (Figure 2, Tables 4 and 5). The DSC thermograms of trastuzumab showed two transitions while ADCs showed at least three transitions (Figure 2A). Moreover, the melting temperature (T_m_) of ADCs was slightly decreased than trastuzumab (Table 4) and there is a significant enthalpy difference between naked trastuzumab compared to conjugated trastuzumab (Table 5). The thermal stability of trastuzumab-MC-VC-PAB-MMAE was also studied with different drug loads (Figure 2B), which showed a decrease in T_m_ when more drug was loaded on the antibody (Table 4).

**Table 4.** Temperature fit parameters of the ADCs

**ADCs**	**T_m_** **Peak-1**	**T_m_** **Peak-2**	**T_m_** **Peak-3**	**T_m_** **Peak-4**
Trastuzumab		64.8°C		79.1°C
T-DM1		62.5°C	74.8°C	78.9°C
Trastuzumab-MCC-maytansinoid (DAR = 4.1)		63.8°C	73.0°C	78.0°C
Trastuzumab-MC-VC-PAB-MMAE (DAR = 2.0)		63.5°C	75.1°C	79.2°C
Trastuzumab-MC-VC-PAB-MMAE (DAR = 4.0)	54.6°C	62.0°C	73.4°C	78.0°C

**Table 5.** Enthalpy fit parameters of the supplied ADC samples

**ADCs**	**ΔH** **Peak-1 (kJ/moL)**	**ΔH** **Peak-2 (kJ/moL)**	**ΔH** **Peak-3 (kJ/moL)**	**ΔH** **Peak-4 (kJ/moL)**
Trastuzumab		660		3,800
T-DM1		334	618	2,454
Trastuzumab-MCC-maytansinoid (DAR = 4.1)		264	684	2,054
Trastuzumab-MC-VC-PAB-MMAE (DAR = 2.0)		370	890	2,300
Trastuzumab-MC-VC-PAB-MMAE (DAR = 4.0)	140	290	740	2,400

The standard deviation in the replicates was < 10% for all reported values, except trastuzumab-MC-VC-PAB-MMAE (DAR = 4.0), trastuzumab

## Discussion

The AlogP method is capable of calculating estimates for most neutral organic compounds possessing C, H, O, N atoms. This prediction is commonly used in drug discovery and the evaluation of hydrophobic interactions in protein-ligand complex [12]. The present results to compare MMAE and maytansinoid were supported by RP-HPLC analysis, therefore this calculation could be considered the use for the structure activity relationship study of MMAE.

The conjugation efficiency of maytansinoid was comparable with MMAE (DAR of trastuzumab-MCC-maytansinoid = 4.1, DAR of trastuzumab-MC-VC-PAB-MMAE = 4.0). The same observation was reported in related studies. To obtain lower DAR ADC (DAR = 1.9), we used lower reaction temperature rather than reducing TCEP equivalent. This modification was previously reported [13], and our current study supported the reproducibility of this conjugation process. RP-HPLC analysis of ADC was used in the present study for assessment of hydrophobicity of ADCs as well as DAR determination. Ideally, a combination of varying analytical methods can be used together to understand the average payload status for un-purified ADCs from a data accuracy perspective [16–18]. However, the high concentrated salt of mobile phases for hydrophobic interaction chromatography (HIC) is known to provide relatively lower peak resolution which has the potential risk to cause overwrapping each peak [19]. Therefore, we selected RP-HPLC for this comparison study. Each DAR compound with drug linker (light chain with drug-linker, heavy chain with drug-linker, heavy chain with 2 drug-linkers, and heavy chain with 3 drug-linkers) showed the same trend in between maytansinoid-based ADC and MMAE-based ADC. The retention time comparison of these ADCs in the HPLC chromatogram showed the hydrophobicity of ADCs was clearly reflected by the drug-linkers. Cysteine-based ADCs were well-separated in the HPLC column while lysine-based ADC like T-DM1 provides complicated and problematic chromatogram. Trastuzumab-MCC-maytansinoid could separate in RP-HPLC column with DAR determination, therefore this molecule may be a potential alternative from chemistry manufacturing and control (CMC) point of view [20].

The hydrophobicity difference between maytansinoid and auristatin did not affect the long-term stability in the present study. SEC analysis after 30 days under 4°C showed the aggregation level of all ADCs was similar to naked trastuzumab. This result implied that this formulation buffer condition (histidine, pH 5.2) sufficiently enables stabilizing the hydrophobic ADCs.

In this study, the effect of conjugation on the higher-order structure of the monoclonal antibody (mAb) was evaluated by DSC. In the case of typical IgG1s, DSC analysis shows the unfolding temperature for each domain (CH2, CH3, and Fab) of antibodies. The DSC analysis of trastuzumab was previously reported showing that the first peak around 65°C corresponded to the CH2 domain [21]; the CH3 and Fab domains overlapped as the second peak around 80°C [21]. The local area around the conjugation site is highly hydrophobic due to drug linkers. This result suggests that the stability of that region was reduced, resulting in a decrease in T_m_. The enthalpy was also decreased post conjugation. Native cysteine conjugation occurs mainly in the CH2 domain, which has a hinge region that contains two reactive disulfide bonds for conjugation. Therefore, the peak corresponding to the CH2 domain (peak-2 in Tables 3 and 4) can be deconstructed [22]. Trastuzumab-MC-VC-PAB-MMAE with DAR = 4 was the most hydrophobic ADC in the present study and the CH2 domain was divided into at least two peaks. The broad peak containing two representative peaks possessed heterogeneous positional isomers originating from conjugation chemistry. The Fab segment was also affected by conjugation with drug-linkers because of the disulfide bond between CL and CH1 domains. This resulted in a new defined peak (peak-3 in Tables 3 and 4), which shifted from the originally combined CH3 and Fab peak in naked antibody. Although the CH3 domain was not expected to be destabilized by conjugation because there were no interchain disulfide bonds to be reduced, the enthalpy of CH3 decreased after conjugation. This result could be attributed to the shift of Fab peaks before and after conjugation. The ADC with lower DAR (trastuzumab-MC-VC-PAB-MMAE with DAR = 1.9) showed slightly higher T_m_ and enthalpy, indicating that this ADC was more stable than the ADC with DAR = 4 but was much less stable than naked trastuzumab. Maytansinoid-based ADC (trastuzumab-MCC-maytansinoid) showed similar results with MMAE-based ADCs. Regardless of the drug-linker, the stability detected by DSC was decreased by conjugation. T-DM1 showed an interesting result that the CH3 domain was not drastically shifted post conjugation [21]. Lysine conjugation to provide T-DM1 occurs in all lysine residues including ones in the CH3 domain; therefore, it was expected that the CH3 of T-DM1 would be affected by conjugation. This observation may be supported by conjugation site analysis of T-DM1 [23]. Conjugation site analysis using peptide mapping was reported by several groups [23–25], and solvent exposure analysis to compare T-DM1 and naked trastuzumab indicated that most modification to produce T-DM1 happened in Fab and CH2 domains [26].

In conclusion, several analytical methods were employed for the characterization at different points in the preparation of ADC which was produced by utilizing different drug-linkers. The comparison of multiple analytical methods using AlogP calculation, RP-HPLC, and DSC allowed for their direct comparison and the evaluation of two major drug-linker-based ADCs. In present studies, maytansinoid-based ADCs showed less hydrophobicity than MMAE-based ADCs, however, regardless of the drug-linker and DARs, the stability detected by DSC was decreased by conjugation.

To precisely evaluate for characterizing these ADCs, several tasks and challenges remain. Several unique drug-linkers were recently published describing a VC-linker-based maytansinoid [27], non-cleavable MMAE [28], and different maytansinoid-based ADCs by cysteine conjugation from our present study [29]. Furthermore, a comparison of the biological properties of ADCs is critical information to be applied for biopharmaceutics. Very recently, our group reported biological studies of maytansinoid-ADCs [10] and MMAE-based ADCs [30] derived from the same antibody by cysteine conjugation chemistry. And further comparison studies including physiological analysis are ongoing. To understand the characteristics of ADCs, the cost and time-efficient analytical comparison described in this manuscript may be useful information for an initial characterization of ADCs prior to detailed biological studies. We are confident that continued innovations and strategies in the field of protein analytical chemistry will aid in the further development of ADC manufacturing.

## Abbreviations

ADC: antibody-drug conjugate
DARs: drug-to-antibody ratios
DSC: differential scanning calorimetry
HC + drug-linker: antibody heavy chain conjugated with one drug-linker
HC: antibody heavy chain
HPLC: high-performance liquid chromatography
LC: antibody light chain
MC: maleimidocaproyl
MCC: (*N*-maleimidomethyl)cyclohexane-1-carboxylate
MMAE: monomethyl auristatin-E
MPB: mobile phase B
PAB: p-aminobenzoyloxycarbonyl
RP-HPLC: reverse phase high-performance liquid chromatography
rt: room temperature
SEC: size exclusion chromatography
TCEP: tris(2-chloroethyl) phosphate
T-DM1: trastuzumab emtansine
TFA: trifluoroacetic acid
T_m_: melting temperature
VC: valine-citrulline

## Supplementary materials

The supplementary material for this article is available at: https://www.explorationpub.com/uploads/Article/file/100264_sup_1.pdf.

## Declarations

### Copyright

© The Author(s) 2021.
