# Supplementary material for: Physical characteristics comparison between maytansinoid-based and auristatin-based antibody-drug conjugates
Source: Explor Target Antitumor Ther. 2021 Dec 31;2(6):576–85. doi: 10.37349/etat.2021.00064 (PMC9400747; doi:10.37349/etat.2021.00064)
Supplement: Supplementary file 1 [file etat-02-100264-s001.pdf]

Supporting Information

Physical characteristics comparison between maytansinoid-based and auristatin-based antibody-drug conjugates

Overview of current approved ADCs..S–2
Chemical structure of each payloads..S–3
Full length chromatograms for HPLC..S–4
DSC analysis..S–8

Overview of current approved ADCs

Table S1. FDA-approved ADCs and their Drug-Linkers

General name	ADC name	Approval	Drug-Linker	Mode of Action
Mylotag®	Gemtuzumab ozogamicin	2000, 2017	calicheamicin	DNA damaging agent
Adcetris®	Brentuximab vedotin	2011	MMAE	microtubule inhibitor
Kadcyla®	Trastuzumab emtansine	2013	DM1	microtubule inhibitor
Besponsa®	Inotuzumab ozogamicin	2017	calicheamicin	DNA damaging agent
Polivy®	Polatuzumab vedotin	2019	MMAE	microtubule inhibitor
Padcev®	enfortumab vedotin-ejfv	2019	MMAE	microtubule inhibitor
Enhertu®	fam-trastuzumab deruxtecan-nxki	2019	Dxd	topoisomerase inhibitor
Trodelvy®	sacituzumab govitecan-hziy	2020	SN-38	topoisomerase inhibitor
Blenrep®	belantamab mafodotin-blmf	2020	MMAF	microtubule inhibitor
Zynlonta™	loncastuximab tesirine-lpyl	2021	tesirine	DNA damaging agent
Tivdak™	tisotumab	2021	MMAE	microtubule inhibitor

	vedotin-tftv

Chemical structure of each payloads

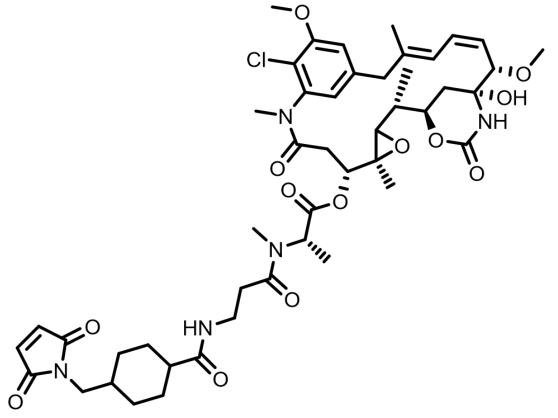

MCC-Maytansinoid

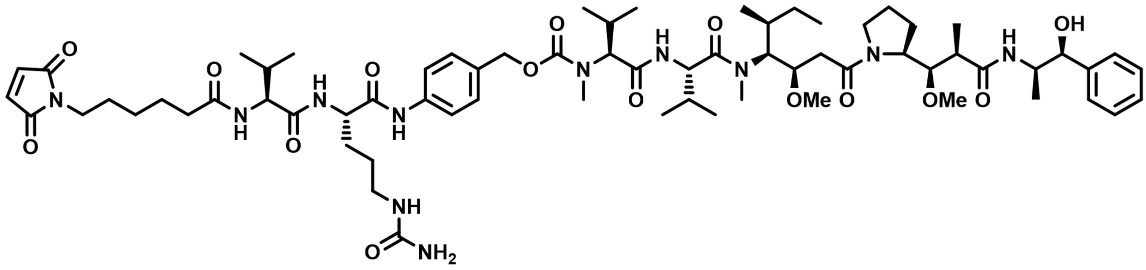

MC-VC-PAB-MMAE

Figure S1. Chemical structure of each payloads

HPLC analysis

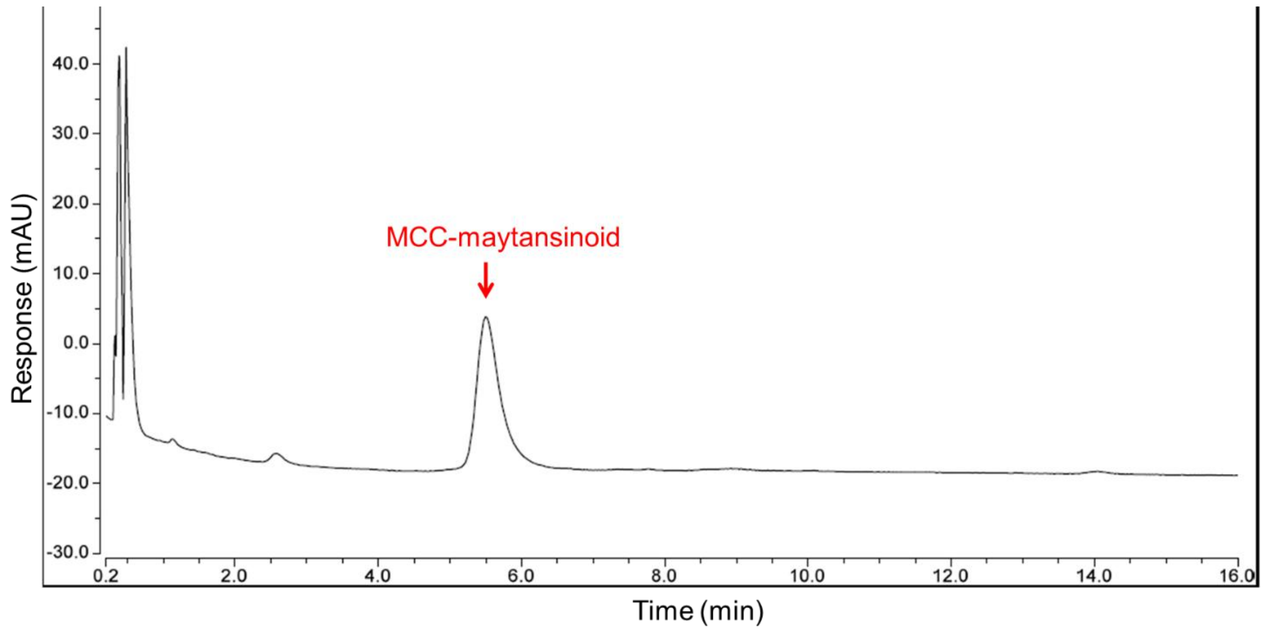

Figure S2. Full length chromatogram for MCC-maytansinoid

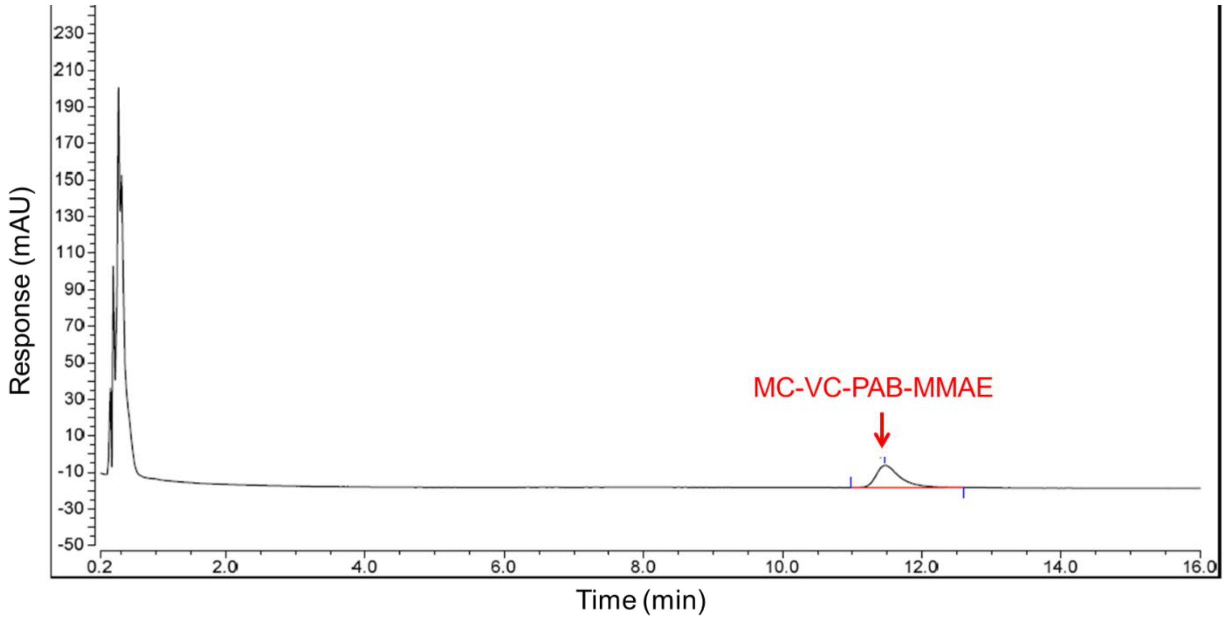

Figure S3. Full length chromatogram for MC-VC-PAB-MMAE

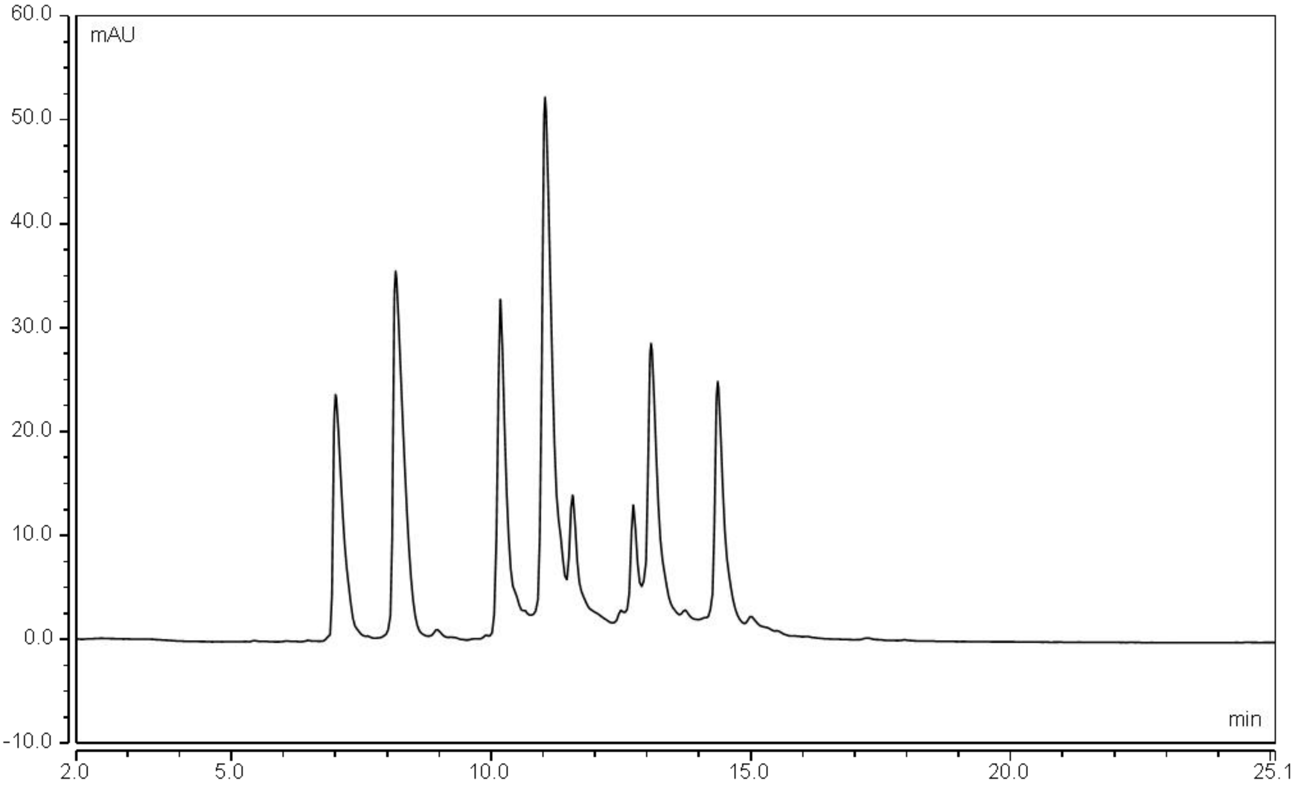

Figure S4. RP-HPLC chromatogram for trastuzumab-MCC-maytansinoid with DAR = 4.1

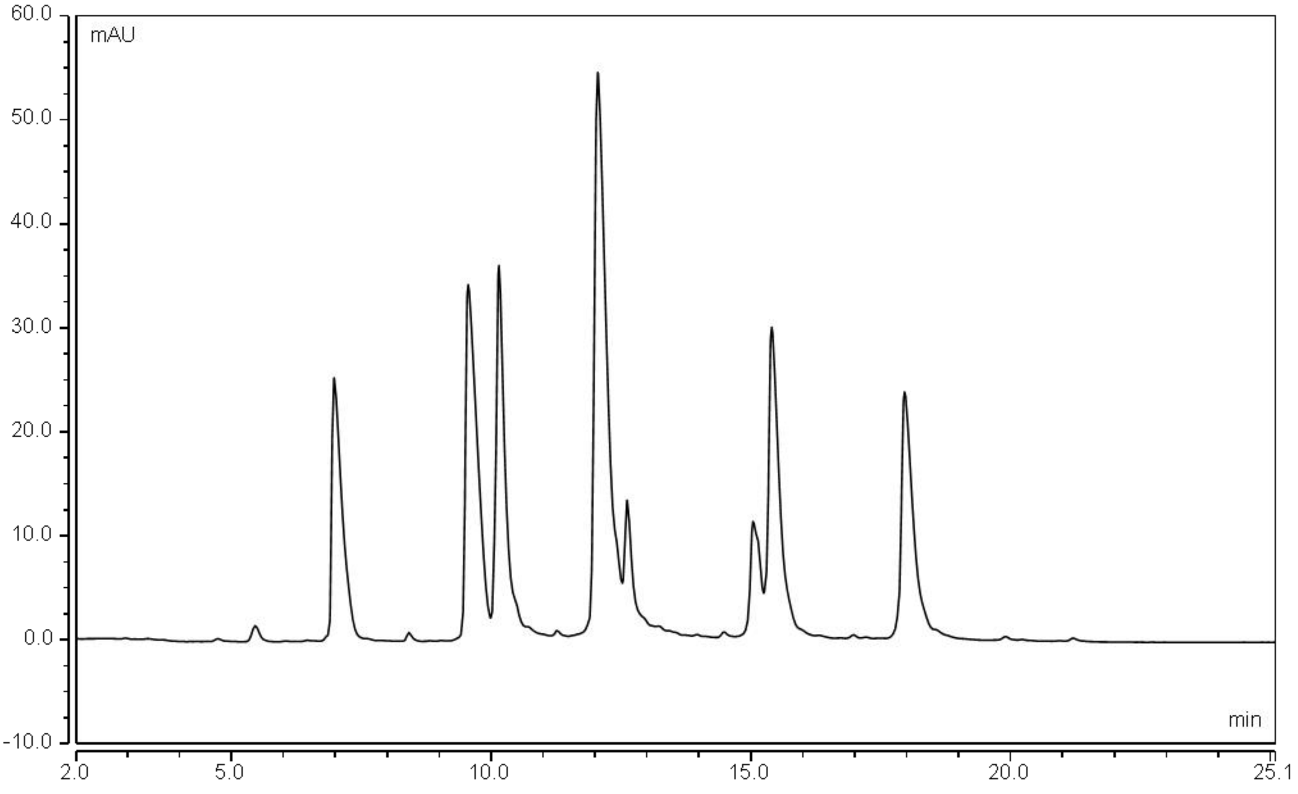

Figure S5. RP-HPLC chromatogram for trastuzumab-MC-VC-PAB-MMAE with DAR 4.0

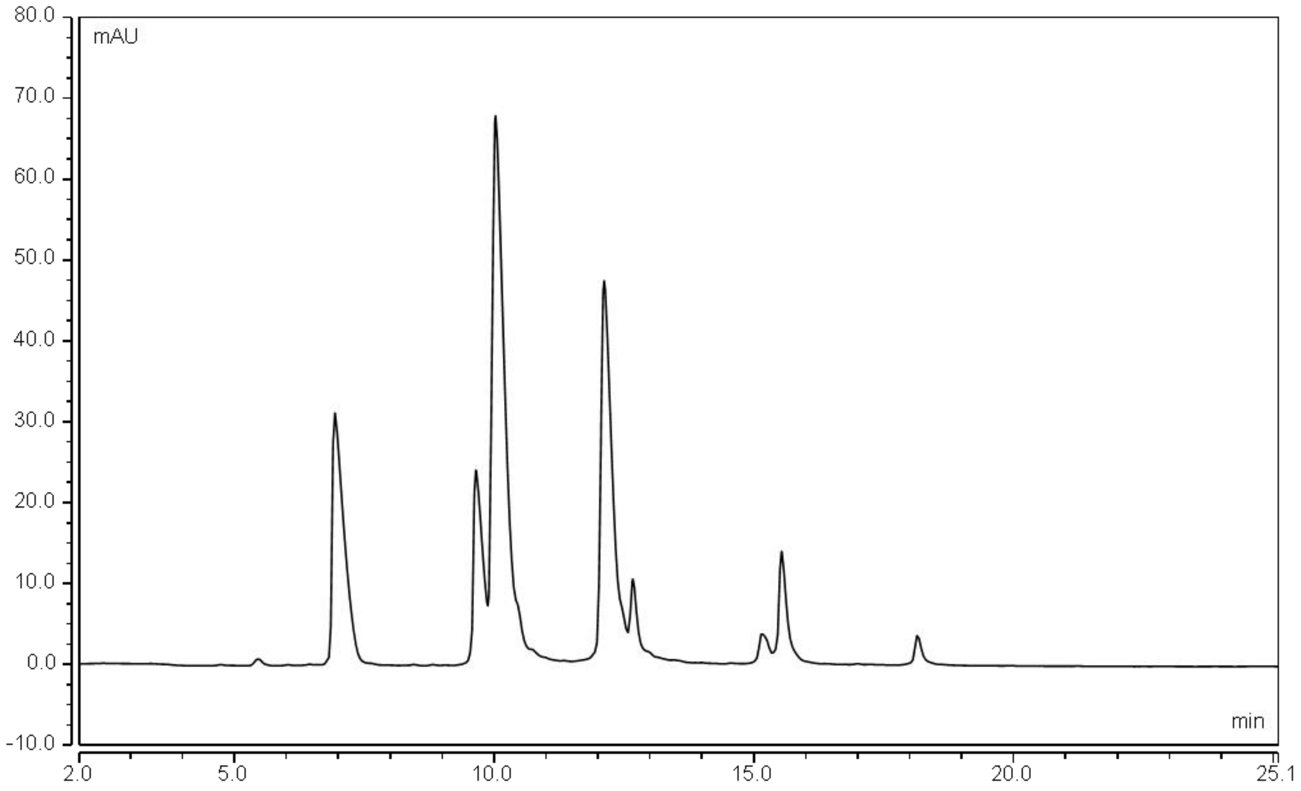

Figure S6. RP-HPLC chromatogram for trastuzumab-MC-VC-MMAE with DAR 1.9

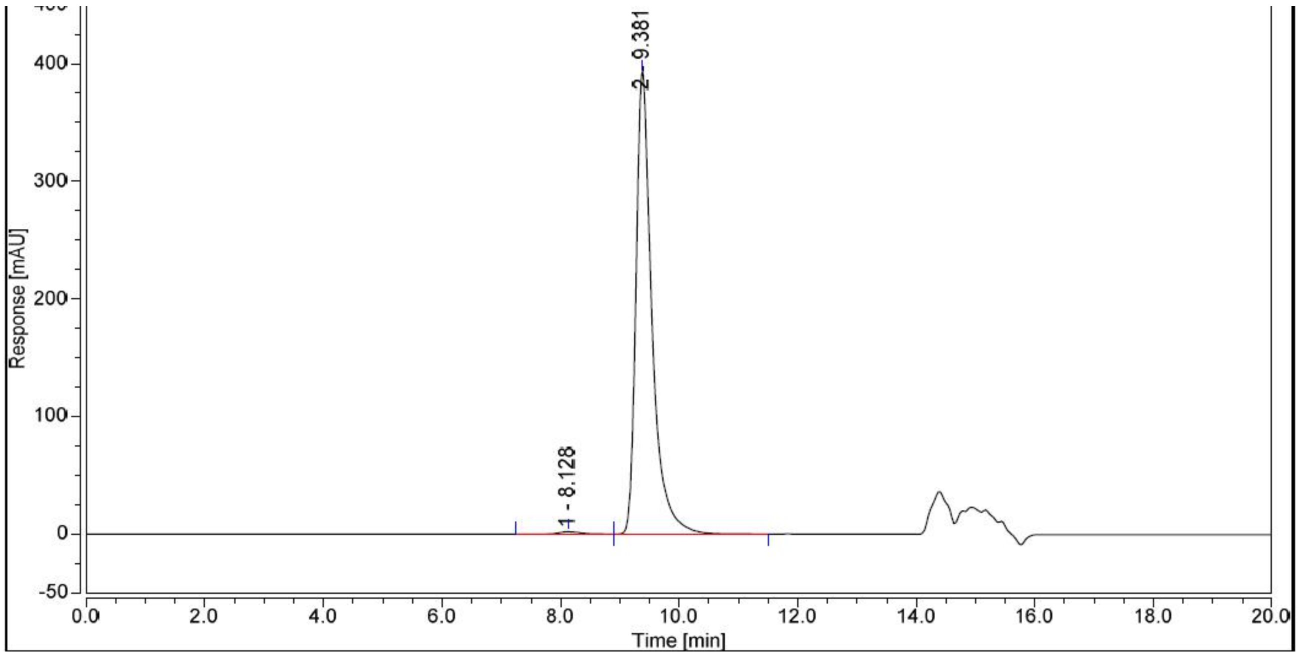

Figure S7. SEC chromatogram for trastuzumab-MCC-maytansinoid with DAR = 4.1

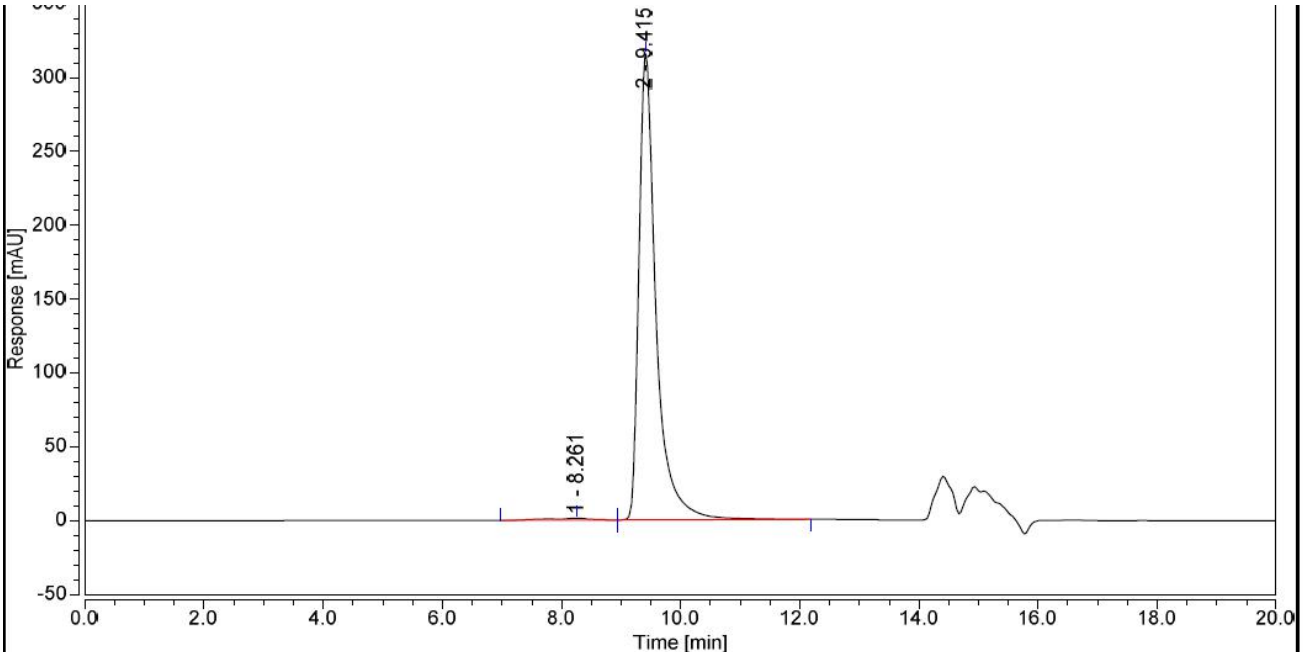

Figure S8. SEC chromatogram for trastuzumab-MC-VC-PAB-MMAE with DAR 4.0

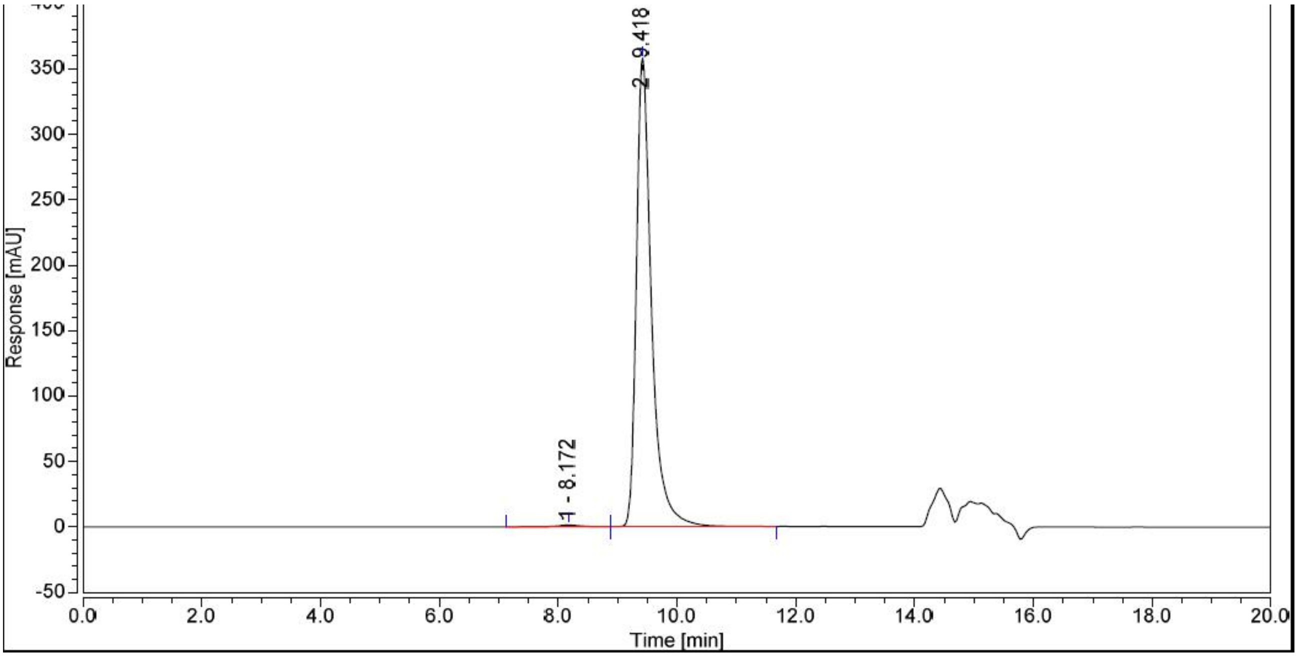

Figure S9. SEC chromatogram for trastuzumab-MC-VC-MMAE with DAR 1.9

DSC analysis

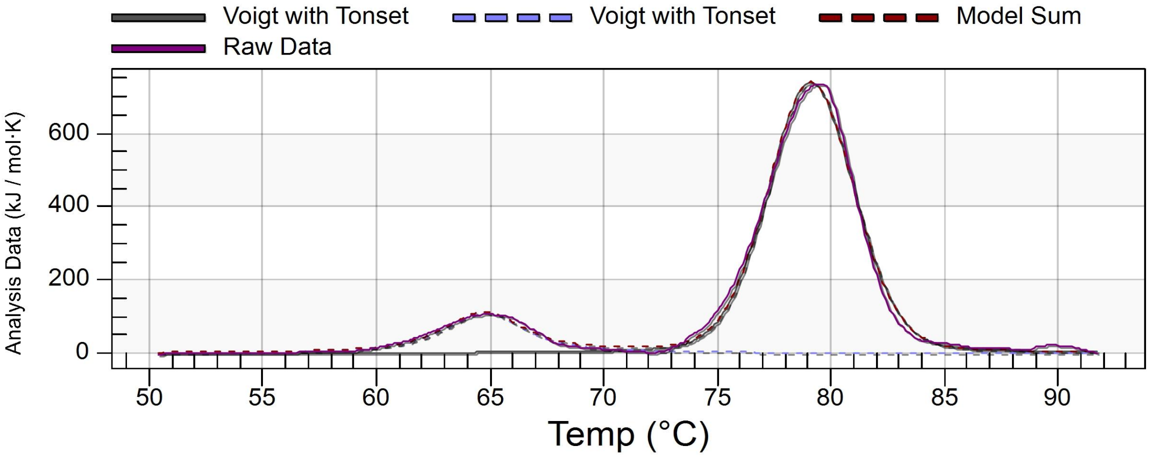

Figure S10. Example of data fitting of trastuzumab

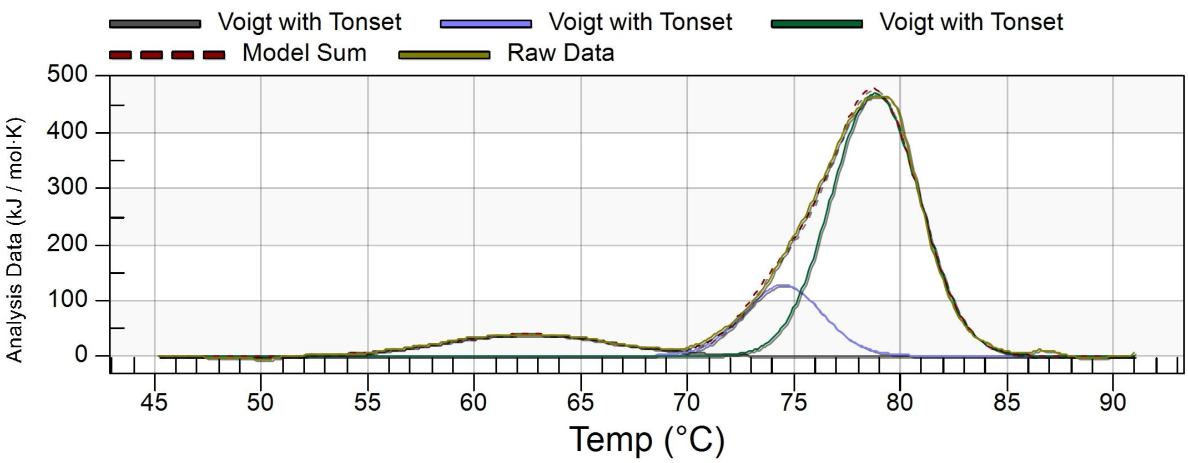

Figure S11. Example of data fitting of T-DM1 (Kadcyla)

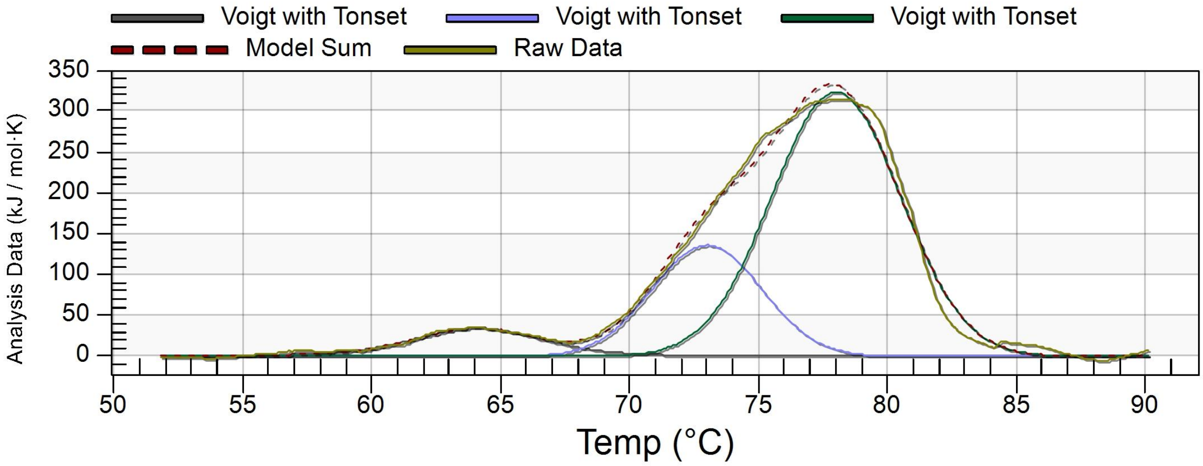

Figure S12. Example of data fitting of trastuzumab-MCC-maytansinoid (DAR = 4.1)

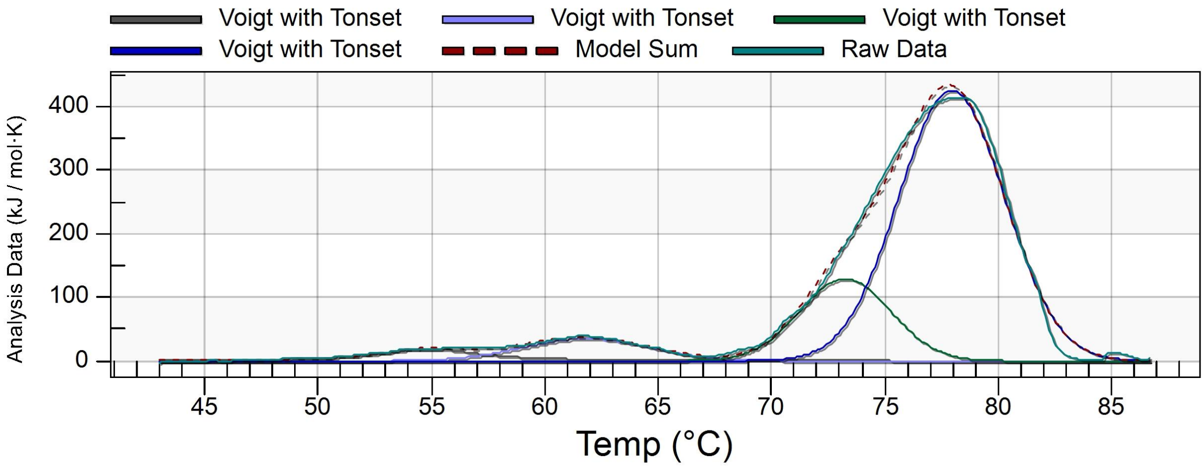

Figure S13. Example of data fitting of trastuzumab-MC-VC-MMAE (DAR = 4.0)

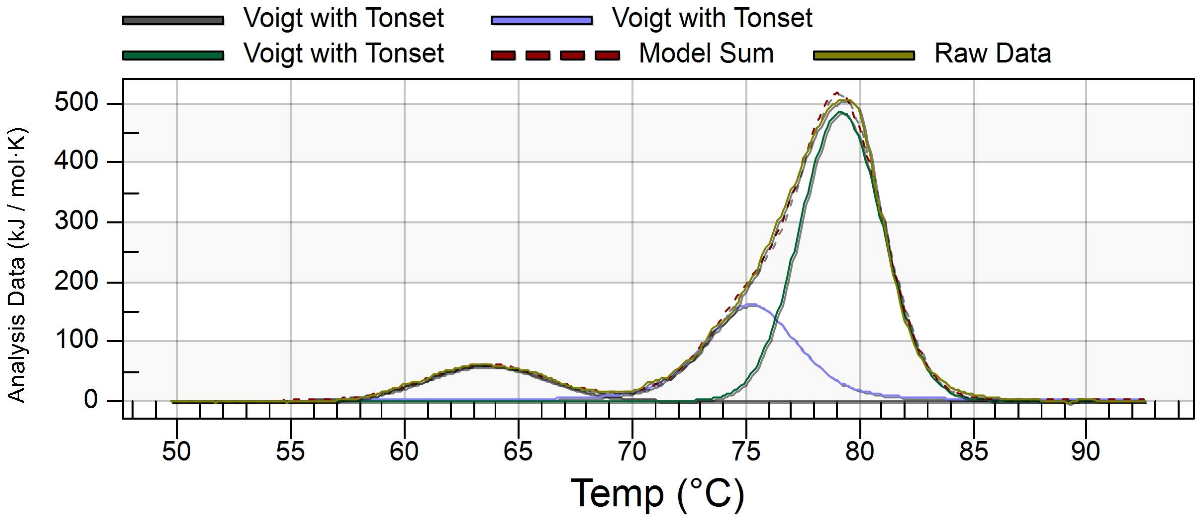

Figure S14. Example of data fitting of trastuzumab-MC-VC-MMAE (DAR = 1.9)
